# Gene Expression Profiles of Human Adipose Tissue-Derived Mesenchymal Stem Cells Are Modified by Cell Culture Density

**DOI:** 10.1371/journal.pone.0083363

**Published:** 2014-01-06

**Authors:** Dae Seong Kim, Myoung Woo Lee, Keon Hee Yoo, Tae-Hee Lee, Hye Jin Kim, In Keun Jang, Yong Hoon Chun, Hyung Joon Kim, Seung Jo Park, Soo Hyun Lee, Meong Hi Son, Hye Lim Jung, Ki Woong Sung, Hong Hoe Koo

**Affiliations:** 1 Department of Pediatrics, Samsung Medical Center, Sungkyunkwan University School of Medicine, Seoul, Korea; 2 Department of Laboratory of Cancer and Stem Cell Biology, Plant Engineering Institute, Sejong University, Seoul, Korea; 3 Top Class plastic surgery, Seoul, Korea; University of California, San Diego, United States of America

## Abstract

Previous studies conducted cell expansion *ex vivo* using low initial plating densities for optimal expansion and subsequent differentiation of mesenchymal stem cells (MSCs). However, MSC populations are heterogeneous and culture conditions can affect the characteristics of MSCs. In this study, differences in gene expression profiles of adipose tissue (AT)-derived MSCs were examined after harvesting cells cultured at different densities. AT-MSCs from three different donors were plated at a density of 200 or 5,000 cells/cm^2^. After 7 days in culture, detailed gene expression profiles were investigated using a DNA chip microarray, and subsequently validated using a reverse transcription polymerase chain reaction (RT-PCR) analysis. Gene expression profiles were influenced primarily by the level of cell confluence at harvest. In MSCs harvested at ∼90% confluence, 177 genes were up-regulated and 102 genes down-regulated relative to cells harvested at ∼50% confluence (*P*<0.05, FC>2). Proliferation-related genes were highly expressed in MSCs harvested at low density, while genes that were highly expressed in MSCs harvested at high density (∼90% confluent) were linked to immunity and defense, cell communication, signal transduction and cell motility. Several cytokine, chemokine and growth factor genes involved in immunosuppression, migration, and reconstitution of damaged tissues were up-regulated in MSCs harvested at high density compared with MSCs harvested at low density. These results imply that cell density at harvest is a critical factor for modulating the specific gene-expression patterns of heterogeneous MSCs.

## Introduction

Human mesenchymal stem cells (MSCs) can be isolated from a wide variety of tissues [1] and are promising candidates for cell-based transplantation and regenerative medicine therapies [2]–[4]. The tendency of MSCs to preferentially home to damaged tissues, their unique immunosuppressive properties [5], and their capacity for self-renewal, combined with multilineage differentiation potential [6], are some of the unique features that make MSCs attractive targets for therapeutic applications. Although the most common and well-characterized source of MSCs is the bone marrow (BM), adipose tissue (AT) is a promising source of MSCs. AT has several advantages as a tissue source, including abundance, easy accessibility, less invasive collection procedures and self-replenishing tendencies [7], [8]. AT-MSCs exhibit the typical immunophenotypes and functional characteristics of MSCs, which is thought to be an alternative source of MSCs for clinical applications than the conventionally used BM.

MSCs exhibit heterogeneous characteristics with regard to morphology, proliferation rate and secreted factors [9]–[13]. Although several clinical trials employing MSCs are currently in progress worldwide, results using MSCs are controversial [14], [15] and may reflect the heterogeneity of MSCs, as well as differences in culture conditions. In particular, *ex vivo* expansion of MSCs is a procedure for developing and maintaining MSCs used for cell therapy and the methods used to expand and characterize the cells are critical factors in preparing MSCs. Moreover, MSCs express a wide variety of cytokines, chemokines and growth factors that are important for cell migration, homing and immunomodulation, following reconstitution of damaged tissues [11], [14], [16]–[18]. Based on their functional effects, the difference in the secretion of these molecules by MSCs could possibly have a critical effect on the results of specific application for cell therapy. In this regards, it is important to identify the best subpopulation of cells and determine how the cells are expanded and characterized *ex vivo* and when they should be used clinically. Numerous attempts have been made to develop more specific procedures for isolation and preparation of appropriate subsets of cells from this heterogeneous cell population. However, protocols for preparing and characterizing MSCs have not yet been standardized. *Ex vivo* expansion of MSCs is one of the alternatives for overcoming the heterogeneity and recent reports suggest that low initial plating densities could be beneficial for optimal *ex vivo* expansion and subsequent differentiation of MSCs [19]–[21].

In this study, we explored the differences in gene expression of AT-MSCs harvested at different cell densities using microarray technology. Cell proliferation genes were highly expressed in MSCs harvested at low density, while genes that were highly expressed in MSCs harvested at high density (∼90% confluent) were linked to immunity and defense, cell communication, signal transduction and cell motility. These results were subsequently validated using RT-PCR. *Ex vivo* expansion of MSCs and harvesting at an adequate cell density could provide a promising strategy for preparing appropriate MSCs to be used in regenerative medicine therapies.

## Results

### Characteristics of AT-derived MSCs and cultures by seeding density

**Figure 1:**
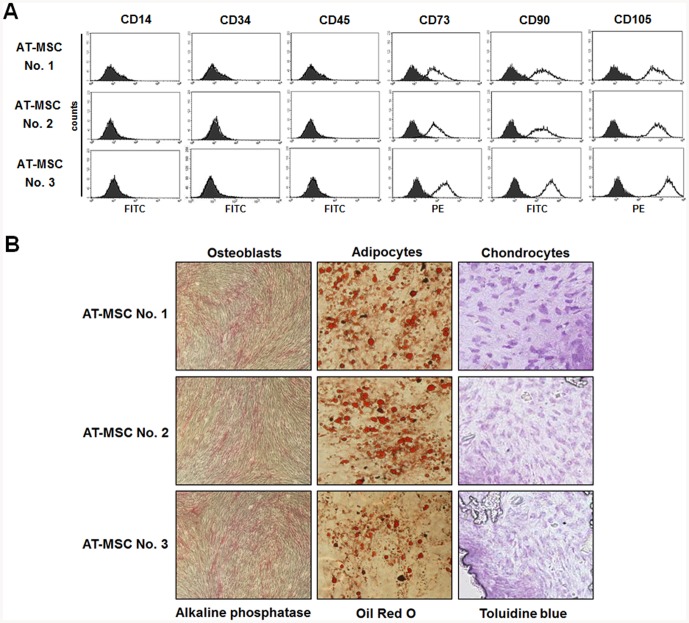
Characterization of AT-MSCs from three different donors. (**A**) The immunophenotype of AT-MSCs from three donors was analyzed by flow cytometry. The expression of surface antigens was plotted against appropriate IgG isotype controls (black histogram). MSCs used for the analyses were positive for CD73, CD90 and CD105, and negative for CD14, CD34 and CD45 (clear histogram). The histograms presented are representative of 3 independent experiments. (**B**) Differentiation of AT-MSCs from three donors. Cells were incubated for 14–21 days in the presence of specific differentiation agents for osteoblasts, chondrocytes, and adipocytes. Alkaline phosphatase staining shows mineralization of the extracellular matrix. Toluidine Blue staining shows the deposition of proteoglycans and lacunae. Differentiation into the adipocyte lineage was demonstrated by staining with Oil Red O. (Magnification: ×100).

Human MSCs were isolated from adult human ATs that were taken from the thigh during cosmetic surgery. The age, weight, and height were shown in Table 1. FACS analysis showed that AT-MSCs derived from three different donors were positive for the typical MSC antigens (CD73, CD90, and CD105) but negative for typical hematopoietic antigens (CD14, CD34, and CD45) (Fig. 1A). Also, expanded cells maintained the potential to differentiate into osteoblasts, adipocytes and chondrocytes (Fig. 1B), indicating that all three populations were comprised of MSCs.

**Table 1:** AT-MSC donor demographics: gender, age, weight, height, and tissue-harvesting site (THS).

Donor	gender	Age (years)	Weight (kg)	Height (cm)	THS
**AT-MSC D1**	Female	31	62	157	Thigh
**AT-MSC D2**	Female	23	51	169	Thigh
**AT-MSC D3**	Female	25	53	161	Thigh

**Figure 2:**
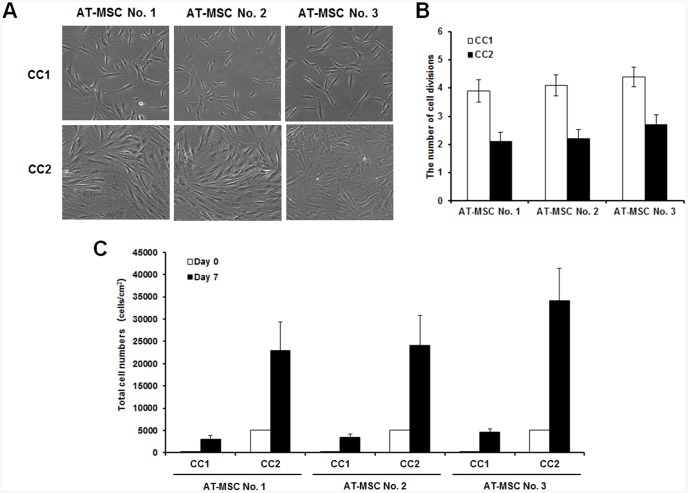
Phase-contrast micrograph and cell density of AT-MSCs from three different donors in CC1 or CC2. (**A**) Morphological appearance of AT-MSC donors 7 days after plating at 200 cells/cm^2^ (CC1) or 5,000 cells/cm^2^ (CC2). All cells exhibited a spindle shaped or fibroblastic morphology. (**B**) The number of cell divisions and (**C**) total cell numbers at the time of harvest of MSCs cultured under different conditions. Data are the mean ± SD from three separate experiments.

AT-MSCs derived from three different donors were plated at a density of 200 (CC1) or 5,000 (CC2) cells/cm^2^ and incubated for 7 days (Fig. 2A). Cultures plated at a density of 200 cells/cm^2^ were ∼50% confluent after 7 days, and most cells were thin and spindle shaped. Cultures plated at 5,000 cells/cm^2^ were ∼90% confluent after 7 days and most cells had extensive cell-to-cell contacts. The number of cell divisions (Fig. 2B) and yields (Fig. 2C) of MSCs from three donors were similar in the same culture condition. However, MSCs from each of three donors showed differences occurring in the proliferation rate between culture conditions. The number of cell divisions of CC1 MSCs from donor No. 1, No. 2 and No. 3 was 3.9±0.40, 4.1±0.37 and 4.4±0.35 after 7 days, respectively, whereas the number of cell divisions of CC2 MSCs from donor No. 1, No. 2 and No. 3 was 2.1±0.32, 2.2±0.34 and 2.7±0.36 after 7 days, respectively. The yields of CC1 MSCs from three donor No. 1, No. 2 and No. 3 was 3,028±800, 3,428±822 and 4,608±772 cells/cm^2^ after 7 days, respectively, whereas the yields of CC2 MSCs from donor No. 1, No. 2 and No. 3 was 22,880±6,434, 24,040±6,803 and 34,173±7,245 cells/cm^2^ after 7 days, respectively. AT-MSCs from donor No. 3 showed the highest proliferation rate and AT-MSCs from donor No. 1 showed the lowest proliferation rate in the same culture.

### Gene expression profiles of AT-MSCs cultured at different seeding densities

**Figure 3:**
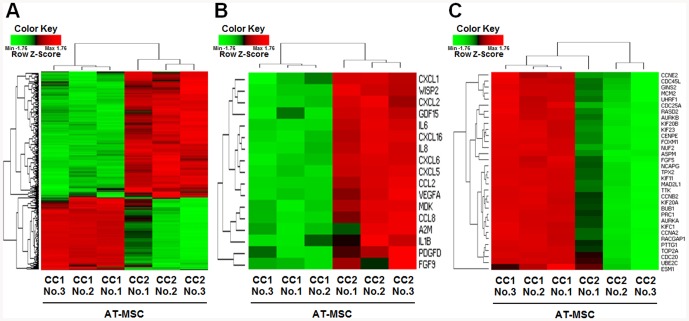
Hierarchical cluster analysis of differentially expressed genes in AT-MSCs from three different donors in CC1 or CC2. The microarray data for 47,323 genes were filtered by applying two criteria for significance, *P*<0.05 and fold change (FC)>2, between the two cell densities (CC1 and CC2) at harvest for each MSC donor. (**A**) The selected data represented by hierarchical clustering of the normalized Ct of 279 genes on MSCs using individual samples (177 with increased expression, 102 with decreased expression). Each row represents a single gene, while each column represents the gene expression levels for a cell culture. The color coded gene expression levels range from red for the highest level of expression to green for the lowest. (**B**) Hierarchical cluster analysis of 17 differentially expressed cytokine, chemokine and growth factor genes. (**C**) Hierarchical cluster analysis of 33 differentially expressed proliferation-associated genes. CC1, cultures plated with an initial cell density of 200 cells/cm^2^ and a culture duration of 7 days; CC2, cultures plated with an initial cell density of 5,000 cells/cm^2^ and a culture duration of 7 days.

Microarray analysis was performed to identify the differentially expressed genes in MSCs plated at different cell densities after harvesting. Gene expression data were compared among AT-MSC cultures obtained from three donors, plated at densities of 200 or 5,000 cells/cm^2^ and cultured for 7 days. Among 47,323 genes analyzed, 279 differentially expressed genes were detected (Table S1). Of differentially expressed genes, 177 genes with increased expression and 102 genes with decreased expression were found in MSCs plated at a density of 5,000 cells/cm^2^ after 7 days of culture (CC2) *vs.* MSCs plated at 200 cells/cm^2^ and examined after 7 days in culture (CC1) (*p*-value<0.05, and >2 fold difference in expression between culture conditions in each of the three MSC donors) (Table S1). Hierarchical clustering analysis of the microarray data revealed differences in gene expression among the six cultures (*p*-value<0.05, and fold change >2) (Fig. 3A). A gene ontology analysis showed that the differentially expressed genes mapped primarily to the categories of molecular and cellular functions such as signal transduction, nuclear metabolism, cell cycle, immunity and defense, cell structure and motility, and cell proliferation. The genes highly expressed in CC2 MSCs were linked to immunity and defense, cell communication, signal transduction and cell motility. Especially, cytokine, chemokine and growth factor genes, including *IL1B*, *IL6*, *A2M*, *MDK*, *CXCL1*, *CXCL2*, *CXCL5*, *CXCL6*, *IL8*, *CXCL16*, *CCL2*, *CCL8*, *WISP2*, *FGF9*, *PDGFD*, *VEGFA* and *GDF15*, were highly expressed in CC2 MSCs compared to CC1 MSCs (Fig. 3B, Table 2). By contrast, the genes highly expressed in CC1 MSCs included factors involved in cell proliferation, cell cycle, nucleic acid metabolism, cell structure. The proliferation genes, including *UBE2C*, *KIF20A*, *NCAPG*, *TPX2*, *BUB1*, *GINS2*, *RACGAP1*, *FOXM1*, *UHRF1*, *MCM2*, *RASD2*, *FGF5*, *CDC25A*, *CCNE2*, *ESM1*, *TOP2A*, *CDC45L*, *AURKA*, *PRC1*, *KIFC1*, *PTTG1*, *AURKB*, *KIF23*, *KIF11*, *KIF20B*, *CENPE*, *ASPM*, *TTK*, *MAD2L1*, *NUF2*, *CDC20*, *CCNA2* and *CCNB2*, were highly expressed in CC1 MSCs compared to CC2 MSCs (Fig. 3C, Table 3).

**Table 2:** Differentially expressed cytokine genes in AT-MSCs from three different donors, cultured to low or high density, as determined by microarray analysis.

			Fold Change		
Gene symbol	Gene description	Gene classification	AT-MSC		
			No. 1	No. 2	No. 3
*Genes up-regulated in CC2 MSCs compared to CC1 MSCs (CC2 MSC/CC1 MSC)*					
***IL1B***	Interleukin-1 beta	Interleukin (immunoglobulin superfamily)	**3.29**	**5.52**	**6.39**
***IL6***	Interleukin-6	Interleukin (Type I cytokine)	**3.37**	**4.75**	**3.55**
***A2M***	alpha-2-Macroglobulin	Other cytokines and related genes	**2.79**	**6.30**	**5.70**
***MDK***	Midkine	Heparin-binding growth factors	**2.58**	**2.75**	**2.50**
***CXCL1***	Chemokine (C-X-C motif) ligand 1	Chemokine (C-X-C motif) ligands	**2.94**	**2.30**	**4.54**
***CXCL2***	Chemokine (C-X-C motif) ligand 2	Chemokine (C-X-C motif) ligands	**5.53**	**6.44**	**4.21**
***CXCL5***	Chemokine (C-X-C motif) ligand 5	Chemokine (C-X-C motif) ligands	**3.26**	**4.54**	**5.32**
***CXCL6***	Chemokine (C-X-C motif) ligand 6	Chemokine (C-X-C motif) ligands	**7.76**	**7.02**	**13.06**
***IL8 (CXCL8)***	Interleukin-8	Chemokine (C-X-C motif) ligands	**10.13**	**11.37**	**14.09**
***CXCL16***	Chemokine (C-X-C motif) ligand 16	Chemokine (C-X-C motif) ligands	**2.04**	**2.11**	**2.26**
***CCL2***	Chemokine (C-C motif) ligand 2	Chemokine (C-C motif) ligands	**5.97**	**9.76**	**14.17**
***CCL8***	Chemokine (C-C motif) ligand 8	Chemokine (C-C motif) ligands	**2.52**	**3.75**	**3.86**
***WISP2***	WNT1-inducible-signaling pathway protein 2	Connective tissue growth factors	**10.41**	**6.57**	**8.10**
***FGF9***	Fibroblast growth factor 9	Fibroblast growth factors	**2.47**	**2.24**	**6.75**
***PDGFD***	Platelet-derived growth factor D	Platelet-derived growth factors	**2.39**	**3.31**	**3.99**
***VEGFA***	Vascular endothelial growth factor A	Vascular endothelial growth factors	**2.00**	**3.09**	**4.53**
***GDF15***	Growth differentiation factor 15	Transforming growth factor beta	**2.00**	**3.98**	**3.49**

Viable second-passage AT-MSCs plated at 200 cells/cm^2^ (CC1 MSCs) or 5,000 cells/cm^2^ (CC2 MSCs) were incubated for 7 days by which time they reached ∼50% or ∼90% confluence, respectively. After harvesting, total mRNA was isolated from pooled samples of MSCs from three donors and used in the microarray analysis. Microarray data were filtered by applying two criteria for significance, P<0.05 and FC>2 between culture conditions.

**Table 3:** Differentially expressed cell proliferation-associated genes in AT-MSCs from three different donors, cultured to low or high density, as determined by microarray analysis.

			Fold Change		
Gene symbol	Gene description	Gene ontology category	AT-MSC		
			No. 1	No. 2	No. 3
*Genes up-regulated in CC1 MSCs compared to CC2 MSCs (CC1 MSC/CC2 MSC)*					
***UBE2C***	Ubiquitin-conjugating enzyme E2C	GO:0007067 _ mitosis	**2.43**	**7.75**	**19.66**
***KIF20A***	Kinesin family member 20A	GO:0000278 _ mitotic cell cycle	**2.29**	**8.33**	**11.94**
***NCAPG***	Non-SMC condensin I complex, subunit G	GO:0051301 _ cell division	**2.81**	**5.61**	**11.70**
***TPX2***	Targeting protein for Xklp2	GO:0007067 _ mitosis	**2.57**	**5.78**	**10.39**
***BUB1***	Budding uninhibited by benzimidazoles 1	GO:0051301 _ cell divisionGO:0000278 _ mitotic cell cycle	**2.12**	**5.62**	**7.36**
***GINS2***	GINS complex subunit 2	GO:0000278 _ mitotic cell cycle	**2.31**	**3.43**	**9.73**
***RACGAP1***	Rac GTPase-activating protein 1	GO:0033205 _ cell cycle cytokinesis	**2.02**	**4.64**	**7.01**
***FOXM1***	Forkhead box M1	GO:0008284 _ positive regulation of cell proliferation	**2.30**	**4.16**	**5.58**
***UHRF1***	Ubiquitin-like with PHD and ring finger domains 1	GO:0007049 _ cell cycleGO:0008283 _ cell proliferation	**2.01**	**2.72**	**7.01**
***MCM2***	Minichromosome maintenance complex component 2	GO:0007049 _ cell cycleGO:0006260 _ DNA replication	**2.04**	**2.98**	**6.09**
***RASD2***	RASD family, member 2	GO:0051897 _ positive regulation of protein kinase B signaling cascade	**2.04**	**3.21**	**5.37**
***FGF5***	Fibroblast growth factor 5	GO:0051781 _ positive regulation of cell division	**2.01**	**2.99**	**3.83**
***CDC25A***	Cell division cycle 25A	GO:0006260 _ DNA replication GO:0000278 _ mitotic cell cycle	**2.18**	**2.21**	**3.38**
***CCNE2***	Cyclin E2	GO:0000278 _ mitotic cell cycle	**2.20**	**2.05**	**7.07**
***ESM1***	Endothelial cell-specific molecule 1	GO:0001558 _ regulation of cell growth	**2.44**	**2.47**	**2.91**
***TOP2A***	Topoisomerase (DNA) II alpha	GO:0006260 _ DNA replication GO:0000278 _ mitotic cell cycle	**2.45**	**6.52**	**10.88**
***CDC45L***	Cell division cycle 45	GO:0006260 _ DNA replication GO:0000278 _ mitotic cell cycle	**2.21**	**3.34**	**11.81**
***AURKA***	Aurora kinase A	GO:0007049 _ cell cycle GO:0007067 _ mitosis	**2.52**	**6.46**	**12.20**
***PRC1***	Protein regulator of cytokinesis 1	GO:0000910 _ cytokinesis	**2.38**	**6.67**	**12.24**
***KIFC1***	Kinesin family member C1	GO:0051301 _ cell division	**2.22**	**6.34**	**12.54**
***PTTG1***	Pituitary tumor-transforming 1	GO:0000278 _ mitotic cell cycle	**2.10**	**6.20**	**11.31**
***AURKB***	Aurora kinase B	GO:0000278 _ mitotic cell cycle	**2.59**	**4.97**	**10.21**
***KIF23***	Kinesin family member 23	GO:0000278 _ mitotic cell cycle	**2.40**	**5.18**	**7.04**
***KIF11***	Kinesin family member 11	GO:0007067 _ mitosis	**2.20**	**4.60**	**7.52**
***KIF20B***	Kinesin family member 20B	GO:0007067 _ mitosis	**2.14**	**3.94**	**6.27**
***CENPE***	Centromere protein E	GO:0000278 _ mitotic cell cycle	**2.12**	**4.44**	**5.39**
***ASPM***	Asp (abnormal spindle) homolog, microcephaly associated	GO:0007067 _ mitosis	**2.32**	**4.71**	**4.58**
***TTK***	TTK protein kinase	GO:0008284 _ positive regulation of cell proliferation	**2.09**	**4.04**	**5.31**
***MAD2L1***	mitotic arrest deficient 2-like 1	GO:0000278 _ mitotic cell cycle	**2.06**	**3.29**	**5.45**
***NUF2***	NUF2, NDC80 kinetochore complex component	GO:0051301 _ cell division GO:0000278 _ mitotic cell cycle	**2.17**	**3.15**	**3.84**
***CDC20***	Cell division cycle 20	GO:0000278 _ mitotic cell cycle	**2.67**	**9.00**	**21.48**
***CCNA2***	Cyclin A2	GO:0000278 _ mitotic cell cycle	**2.46**	**6.66**	**11.28**
***CCNB2***	Cyclin B2	GO:0000278 _ mitotic cell cycle	**2.15**	**6.23**	**9.16**

Viable second-passage AT-MSCs plated at 200 cells/cm^2^ (CC1 MSCs) or 5,000 cells/cm^2^ (CC2 MSCs) were incubated for 7 days, by which time they reached ∼50% or ∼90% confluence, respectively. After harvesting, mRNA from three donor pooled samples of AT-MSCs was used in the microarray analysis. Microarray data were filtered by applying two criteria for significance, P<0.05 and FC>2 between culture conditions.

### RT-PCR confirmation of microarray data

**Figure 4:**
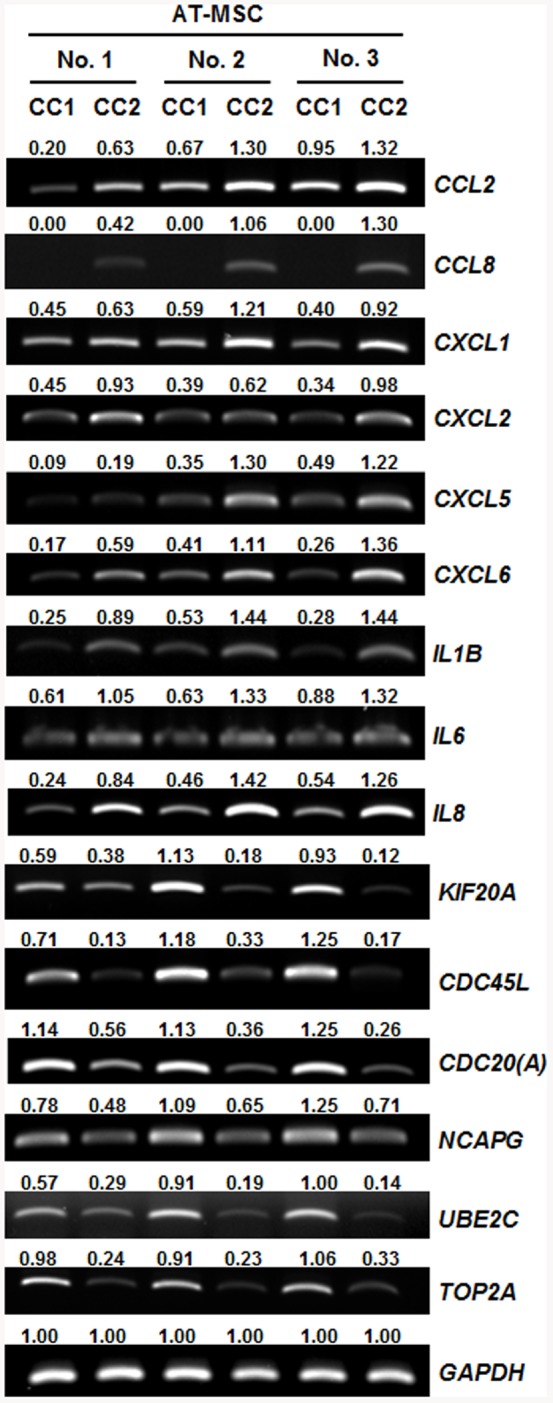
RT-PCR analysis of differentially expressed cytokine, chemokine and proliferation-associated genes in AT-MSC from different donors and different cell densities. The expression profile of selected genes from the microarray data was validated by semi-quantitative RT-PCR using independent samples harvested 7days after plating at different cell densities as distinct from that for microarray analysis. Quantitative gene expression data of each candidate gene indicates mRNA expression relative to GAPDH mRNA. Band intensity was normalized against that of GAPDH mRNA. Semi-quantitative RT-PCR analysis was independently performed using different MSC samples but the samples for microarray analysis. CC1, cultures plated with an initial cell density of 200 cells/cm^2^ and a culture duration of 7 days; CC2, cultures plated with an initial cell density of 5,000 cells/cm^2^ and a culture duration of 7 days.

To validate the cytokine, chemokine and proliferation-associated gene expression profiles determined by microarray analysis, we conducted semi-quantitative RT-PCR, using independent samples of AT-MSC donor cells harvested 7 days after plating at different cell densities as distinct from that for microarray analysis. The differential gene expression pattern observed among the six MSC cultures in the microarray analysis was in good agreement with RT-PCR data for the few select genes tested. Quantitative comparison of the intensity of the detected bands showed that the cell density at harvest was a critical factor correlated with the difference in gene expression in the cultures (Fig. 4). The expression of cytokines and chemokines, including *CCL2*, *CCL8*, *CXCL1*, *CXCL2*, *CXCL5*, *CXCL6*, *IL1B*, *IL6* and *IL8*, was up-regulated in the three AT-MSC donor cultures harvested at high density (CC2, ∼90% confluent) compared to cultures from the same donors harvested at low density (CC1, ∼50% confluent). By contrast, the expression of proliferation-associated genes, including *KIF20A*, *CDC45L*, *CDC20*, *NCAPG*, *UBE2C* and *TOP2A*, was up-regulated in the three AT-MSC donor cultures harvested at low density (CC1, ∼50% confluent) compared cultures from the same donors harvested at high density (CC2, ∼90% confluent).

## Discussion

Human MSCs are attractive candidates for transplantation therapies because they can be easily harvested and expanded, have the potential for autologous transplantation, and home to sites of injury [22], [23]. Despite these desirable features of MSCs, numerous studies and clinical trials using MSCs have yielded conflicting results. This variability may reflect differences between individual donors or differences in the isolation, culture and expansion methods used by individual investigators. According to the International Society for Cellular Therapy (ISCT, 2006), MSCs were defined as follows: (1) adherence to plastic under standard culture conditions; (2) positive for expression of CD105, CD73 and CD90 and negative for expressions of hematopoietic cell surface markers CD34, CD45, CD11a, CD19 or CD79a, CD14 or CD11b and histocompatibility with locus antigen (HLA)-DR; and (3) under a specific stimulus, differentiation into osteocytes, adipocytes and chondrocytes *in vitro*. Although some studies reported that a few early passage AT-MSCs were positive for CD34 [24], [25], those reports are not consistent with representative characteristics of the understating of MSCs at ISCT, 2006 [26], [27]. These discrepancies on the CD34 expression of AT-MSCs among the reported studies may be caused by isolated cells that have different cell composition. Isolated primary cells from adipose tissue contain various cells including CD34^+^ progenitor, stromal cell-like, pericyte-like and endothelial cells as well as AT-MSCs [25], [27], [28]. Cell composition may affect the differences in isolation methods, culture conditions and the human factor, which determines when and how cultures are passaged. Thus, some of cells that are positive for CD34 during early passage may be different types of progenitor cells, and not AT-MSCs. In this study, we used AT-MSCs that were positive for the typical MSC surface antigens (CD73 (SH-2, SH-3), CD90, CD105 and CD166) but negative for hematopoietic lineage antigens (CD14, CD34 and CD45), indicating that this study was conducted using AT-MSCs excluding different types of progenitor cells. Moreover, initial plating densities, level of confluence at harvest, and time of culture are all potentially confounding factors with unknown impacts on therapeutic efficacy. Because MSCs are selected only for their ability to grow under the conditions selected by the investigator, differences in culture conditions could further amplify existing variations. Therefore, in order for MSCs to be used successfully in transplantation, it will be necessary to identify suitable methods for cell expansion in cultures and to characterize the resulting gene-expression patterns.

In this study, we identified genes that were differentially expressed in MSCs from three AT-MSC donors cultured for 7 days after plating at different cell densities. Microarray data revealed that proliferation-related genes were highly expressed in all MSCs harvested at low density, whereas genes associated with the cells' therapeutic functions were highly expressed in all MSCs harvested at high density. Our previous report showed that the genes highly expressed in BM-MSCs harvested at high density largely corresponded to chemotaxis, inflammation, and immune responses, indicating direct or indirect involvement in the immunomodulatory functions [29]. Although differences in gene expression of cytokines, chemokines and growth factors between BM-MSCs and AT-MSCs among cultures harvested at different cell densities did not perfectly coincide due to their different tissue origin (Table S2), all MSCs derived from both BM and AT represented significant differences in the levels of functional gene expression by the cell density at harvest. In contrast, proliferation-related genes were highly expressed in BM-MSCs harvested at low density similar to the data from AT-MSCs (Table S3). Thus, our results suggest the possibility of modulating MSC gene-expression patterns by manipulating cell density, as well as the feasibility of designing a strategy for preparing suitable MSCs using specific culture conditions, in spite of their heterogeneity from different tissue origins and donors.

Previous studies suggest that low initial plating densities result in faster expansion, leading to higher MSC yields [20], [21]. Because growth at higher densities becomes constrained by density-dependent growth inhibition, cells plated at lower densities yield more doublings per passage. In this study, MSCs harvested at low density (CC1 MSCs) exhibited higher proliferation rates than MSCs harvested at high density (CC2 MSCs). The increased rate of cell division in MSCs harvested at low density was consistent with the higher expression of genes involved in proliferation, including *KIF20A*, *CDC45L*, *CDC20A*, *NCAPG*, *UBE2C* and *TOP2A*. These results imply that the growth patterns of MSC cultures depend on cell density. At lower plating density, MSCs dispersed evenly across the plate and, over time, rapidly grew to fill the available space. As they progressed to confluence, their proliferation slowed due to cell-to-cell contact, also reflected in reduced expression of proliferation-related genes. However, the influence of these gene-expression levels on the proliferation potential of MSCs must be investigated further.

MSCs exhibit differences in their functional characteristics, including homing and regenerative potential, because even MSCs from an individual donor exhibit substantial heterogeneity [5], [9]–[13], [16], [19]. A comparison of these properties among MSCs derived from different donors is critical for determining which MSC populations are appropriate for a specific clinical use. In addition, although researchers believe that low initial plating densities are beneficial for the optimal *ex vivo* expansion and subsequent differentiation of MSCs [19]–[21], there is no clear evidence that this specific condition leads to greater efficacy in animal models of disease and injury. In this study, we also identified differences in expression of cytokines, chemokines and growth factors in AT-MSCs among cultures harvested at different cell densities. MSCs harvested at high density (CC2 MSCs) exhibited higher expression of mRNAs encoding secreted factors involved in immunosuppression, migration, homing, angiogenesis and reconstitution of damaged tissues, than MSCs harvested at low density (CC1 MSCs). Factors secreted by MSCs are thought to be important for the therapeutic action of MSCs, and previous studies have attempted to investigate their functions [18], [30]. CXC chemokine genes, including *CXCL1*, *CXCL2*, *CXCL5*, *CXCL6*, *CXCL8* (*IL8*) and *CXCL16*, are up-regulated in CC2 MSCs; these factors mediate angiogenic activity [31] as well as survival, proliferation and chemotaxis of endothelial cells and activated T cells via autocrine and paracrine signaling mechanisms [10], [11], [22], [23], [32], [33]. IL-1B and IL6, which are also highly expressed by MSCs harvested at high densities, are important mediators of the inflammatory response that are involved in a variety of cellular activities including proliferation and differentiation [34]–[36]. CCL2 and CCL8 mediate homing, angiogenic responses, and tissue repair; mildly elevated levels of IL1B and CCL2 stimulate MSC homing and promote repair of damaged tissues under physiological conditions [37]–[39]. In our comparative study of factors secreted by MSCs cultured under different conditions, we detected higher expression of several growth factors that are directly or indirectly associated with angiogenesis, cell migration, proliferation, differentiation, survival and anti-inflammatory effects, including VEGFA [40]–[42], FGF9 [42], PDGFD [43], A2M [44], MDK [45], WISP2 [46] and GDF15 [47], in MSCs harvested at high density. Inflammatory responses, cell migration and angiogenesis are fundamental to the repair of damaged tissues. Therefore, MSCs harvested at high density may be useful in the treatment of conditions associated with damaged tissues, e.g., in graft-versus-host disease, heart infarction and ischemia.

Based on our results, manipulation of cell-culture conditions represents a promising strategy for preparation of MSCs suitable for specific applications. Seeding at low initial density and continuous subconfluent culture helps to reduce selective pressures that may alter cellular composition and function, as well as to obtain sufficient yields of cells for use in treatments. By contrast, continuous confluent cell culture causes morphological changes, reduced proliferation rate and differentiation potential, and even senescence of MSCs [48]–[50]. For this reason, culture of MSCs at confluence, after a sufficient number of cells had been obtained via low-cell density culture, represents an effective method for overcoming those problems. Furthermore, given the heterogeneity of MSCSs as well as their differential responses to culture conditions, such a strategy could influence the therapeutic properties of MSCs. In this study, culture conditions mainly modulated the gene-expression patterns of MSCs; nonetheless, we were able to identify relative differences in the levels of gene expression from different donors under the same culture condition. Therefore, the heterogeneity among MSCs derived from different donors needs to be considered as another critical factor in the selection of potent MSCs. Because AT-MSCs from donor No. 3 expressed the highest level of functional genes among the three MSC donors, we consider that AT-MSCs from donor No. 3 may be suitable for repair of damaged tissues, in both experimental and clinical settings.

In conclusion, this is the first report evaluating the gene-expression profiles of AT-MSCs harvested at different cell densities. Genes highly expressed in MSCs harvested at low density (∼50% confluent) included factors involved in cell proliferation, whereas genes highly expressed in MSCs harvested at high density (∼90% confluent) were associated with the cells' therapeutic functions. These results imply that cell density at harvest is a critical factor for modulating the specific gene-expression patterns of heterogeneous MSCs.

## Materials and Methods

### Isolation and culture of human adipose tissue-derived MSCs (AT-MSCs)

The Institutional Review Board of the Samsung Medical Center approved this study (IRB No. 2009-09-033) and all samples were obtained with written informed consent. Adipose tissues were taken from the thigh region during cosmetic surgery; 3 female donors were included in this study. AT-MSCs were isolated and cultured according to a previous protocol [51]. Briefly, adipose tissues were washed extensively with equal volumes of Dulbecco's phosphate-buffered saline (DPBS; HyClone, Logan, UT), and the extracellular matrix was digested with 0.075% collagenase A (Roche Applied Science, Penzberg, Germany) at 37°C for 30 min. Enzyme activity was neutralized with low glucose Dulbecco's modified Eagle's medium (LG-DMEM; Invitrogen-Gibco, Rockville, MD) containing 10% fetal bovine serum (FBS; Invitrogen-Gibco) and 100 U/mL penicillin/streptomycin (PS; Invitrogen-Gibco). Samples were then centrifuged at 805 *g* for 10 min. The cell pellet was washed with DPBS and filtered through a 100-µm nylon mesh (Cell strainer; Becton Dickinson, Franklin Lakes, NJ). Cells were seeded on uncoated T25 culture flasks (Nalge Nunc, Naperville, IL) at a density of 3×10^5^ cells/cm^2^ in LG-DMEM containing 10% FBS and 100 U/mL PS. The cells were incubated in a humidified atmosphere at 37°C with 5% CO_2_, and the medium was changed every 3 to 4 days until the adherent fibroblast-like cells reached ∼70% confluence.

### Characterization of MSCs by immunophenotypic analysis

Antibodies against the human antigens CD14, CD34, CD45, CD73 and CD90 were purchased from Becton Dickinson. Antibodies against CD105 were purchased from Ancell (Bayport, MN). A total of 5×10^5^ cells were resuspended in 0.2 mL DPBS and incubated with fluorescein isothiocyanate (FITC)- or phycoerythrin (PE)-conjugated antibodies for 30 min at room temperature. The fluorescence intensity of the cells was evaluated by flow cytometry (FACScan; Becton Dickinson) and the data were analyzed using the CELLQUEST software (Becton Dickinson).

### Differentiation of AT-MSCs

#### Osteogenic differentiation

Cells were plated at 5×10^5^ cells/well in 6-well plates in LG-DMEM containing 10% FBS, allowed to adhere overnight, and replaced with LG-DMEM containing 10% FBS supplemented with 0.1 µM dexamethasone (Sigma-Aldrich, St. Louis, MO), 10 mM β-glycerolphosphate (Sigma-Aldrich), and 100 µM ascorbate-2-phosphate (Sigma-Aldrich). The medium was changed every 3 days. After 14–21 days, osteoblast differentiation was determined by alkaline phosphatase expression.

#### Adipogenic differentiation

As described above, cells were cultured for 14–21 days after reaching confluence in LG-DMEM containing 10% FBS, 1 µM dexamethasone, 500 µM isobutyl methylxanthine (Sigma-Aldrich), 100 µM indomethacin (Sigma-Aldrich), and 10 µg/mL insulin (Sigma-Aldrich). Adipogenic differentiation was evaluated by detecting cellular accumulation of neutral lipid vacuoles via staining with Oil-red O (Sigma-Aldrich) solution.

#### Chondrogenic differentiation

A total of 1×10^6^ cells were pelleted in a 15-mL tube by centrifugation at 300 *g* for 5 min. Pelleted cells were cultured for 14–21 days after reaching confluence in LG-DMEM containing 1× insulin-transferrin-selenium (ITS; Invitrogen-Gibco), 1 mM sodium pyruvate (Invitrogen-Gibco), 0.1 µM dexamethasone, 397 µg/mL ascorbate-2-phosphate, and 10 ng/mL transforming growth factor-β1 (R&D Systems, Minneapolis, MN). Chondrogenic induction was evaluated at 80% confluence by detecting extracellular accumulation of chondrocyte matrix by toluidine blue (Sigma-Aldrich) staining.

### Phase contrast microscopy and cell count

Viable second-passage MSCs were used throughout these studies. In this study, culture condition 1 (CC1) refers to cultures plated with an initial cell density of 200 cells/cm^2^ and a culture duration of 7 days, whereas CC2 refers to cultures plated at 5,000 cells/cm^2^, and cultured for 7 days. CC1 and CC2 MSCs were observed on day 7 of culture using a phase contrast microscope (Olympus CK40, Melville, NY). Confluence was estimated by phase contrast microscopy based on the occupied surface of the tissue culture flask and the total cell number was measured by hemocytometer (Marienfeld, German) counts.

### RNA isolation and microarray analysis

Total RNA from CC1 and CC2 MSCs was isolated to perform gene expression profiling. Cultured MSCs were collected by treatment with 0.05% trypsin-EDTA and total cellular RNA was extracted from pelleted cells and purified using a QIAGEN RNeasy Mini Kit (Qiagen, Valencia, CA) according to the manufacturer's protocol. RNA quality was determined using denaturing gel electrophoresis, the OD 260/280 ratio, and analysis on an Agilent 2100 Bioanalyzer (Agilent Technologies, Palo Alto, CA). Biotinylated complementary RNA was prepared and hybridized to the Illumina Human HT-12 Expression Beadchip (Illumina, San Diego, CA). The arrays were scanned and analyzed using the Illumina Genome Studio v2009.2 software (Gene Expression Module v1.5.4, Illumina). The false discovery rate was controlled by adjusting *P*-values by means of the Benjamini-Hochberg algorithm, followed by performance of a Gene Set Enrichment Analysis and a one-tailed Fisher's exact test. The microarray data for 47,323 genes were filtered by applying two criteria for significance, *P*<0.05 and fold change (FC)>2.

### Reverse transcription polymerase chain reaction analysis

Independent samples of AT-MSC donor cells as distinct from that for microarray analysis harvested 7 days after plating at different cell densities. Total RNA from CC1 and CC2 MSCs was isolated using a QIAGEN RNeasy Mini Kit and used to perform semi-quantitative reverse transcription polymerase chain reaction (RT-PCR) assays with a commercial kit (PrimeScript™ 1^st^ strand cDNA synthesis kit; TaKaRa Shuzo, Shiga, Japan). Band intensity was measured using NIH image/ImageJ and normalized against that of GAPDH mRNA. The cDNAs were amplified using the primers shown in Table S4.

### Statistical analysis

All results are expressed as means ± standard deviation (± SD).

## Supporting Information

Table S1 **List of genes that were differentially expressed in MSCs harvested at high cell density (CC2 MSCs, ∼90% confluence) relative to low cell density (CC1 MSCs, ∼50% confluence) from three donors.** Fold changes indicate gene expression differences between MSCs harvested at high cell density (CC2 MSCs, ∼90% confluent) and at low cell density (CC1 MSCs, ∼50% confluent). Viable second-passage AT-MSCs plated at 200 or 5,000 cells/cm2 were incubated for 7 days to approximately 50% confluence or 90% confluence, respectively. Positive values indicate higher expression and negative values indicate lower expression, in MSCs harvested at high cell density relative to low cell density. p<0.05.
(DOC)

Table S2 **Differentially expressed cytokine genes in BM-MSCs, cultured to low or high density, as determined by microarray analysis.** Viable second-passage BM-MSCs plated at 200 cells/cm^2^ (CC1 MSCs) or 5,000 cells/cm^2^ (CC2 MSCs) were incubated for 7 days by which time they reached ∼50% or ∼90% confluence, respectively. After harvesting, total mRNA was isolated from pooled samples of MSCs from three donors and used in the microarray analysis. Microarray data were filtered by applying two criteria for significance, P<0.05 between culture conditions. *n.d*, not detected.
(DOC)

Table S3 **Differentially expressed cell proliferation-associated genes in BM-MSCs, cultured to low or high density, as determined by microarray analysis.** Viable second-passage BM-MSCs plated at 200 cells/cm^2^ (CC1 MSCs) or 5,000 cells/cm^2^ (CC2 MSCs) were incubated for 7 days, by which time they reached ∼50% or ∼90% confluence, respectively. After harvesting, mRNA from pooled samples of BM-MSCs was used in the microarray analysis. Microarray data were filtered by applying two criteria for significance, P<0.05 between culture conditions. *n.d*, not detected.
(DOC)

Table S4 **Primer sequences used for RT-PCR.** *CCL2*, *Chemokine (C-C motif) ligand 2*; *CCL8*, *Chemokine (C-C motif) ligand 8*; *CXCL1*, *Chemokine (C-X-C motif) ligand 1*; *CXCL2*, *Chemokine (C-X-C motif) ligand 2*; *CXCL5*, *Chemokine (C-X-C motif) ligand 5*; *CXCL6*, *Chemokine (C-X-C motif) ligand 6*; *IL1B*, *Interleukin-1, beta*; *IL6*, *Interleukin-6*; *IL8*, *Interleukin-8*; *KIF20A*, *Kinesin family member 20A*; *CDC45L*, *Cell division cycle 45*; *CDC20*, *Cell division cycle 20*; *NCAPG*, *Non-SMC condensin I complex, subunit G*; *UBE2C*, *Ubiquitin-conjugating enzyme E2C*; *TOP2A*, *Topoisomerase II alpha*; *GAPDH*, *glyceraldehyde-3-phosphate dehydrogenase*. ^a^Forward (F) and reverse (R) primers used to detect mRNA expression of the indicated targets.
(DOC)
