# Supplementary material for: Gene Expression Profiles of Human Adipose Tissue-Derived Mesenchymal Stem Cells Are Modified by Cell Culture Density
Source: PLoS One. 2014 Jan 6;9(1):e83363. doi: 10.1371/journal.pone.0083363 (PMC3882209; doi:10.1371/journal.pone.0083363)
Supplement: Table S1 — List of genes that were differentially expressed in MSCs harvested at high cell density (CC2 MSCs, ∼90% confluence) relative to low cell density (CC1 MSCs, ∼50% confluence) from three donors. Fold changes indicate gene expression differences between MSCs harvested at high cell density (CC2 MSCs, ∼90% confluent) and at low cell density (CC1 MSCs, ∼50% confluent). Viable second-passage AT-MSCs plated at 200 or 5,000 cells/cm2 were incubated for 7 days to approximately 50% confluence or 90% confluence, respectively. Positive values indicate higher expression and negative values indicate lower expression, in MSCs harvested at high cell density relative to low cell density. p<0.05. (DOC) [file pone.0083363.s001.doc]

**Table S1. List of genes that were differentially expressed in MSCs harvested at high cell density (CC2 MSCs, ~90% confluence) relative to low cell density (CC1 MSCs, ~50% confluence) from three donors.**

|  |  | **Fold Change** | | |
| --- | --- | --- | --- | --- |
| **Gene Symbol** | **Gene Description** | **AT-MSC** | | |
|  |  | **No.1** | **No.2** | **No.3** |
| ***RARRES2*** | Retinoic acid receptor responder (tazarotene induced) 2 | 9.15 | 11.71 | 17.93 |
| ***IL8*** | Interleukin 8 | 10.13 | 11.37 | 14.09 |
| ***CXCL6*** | Chemokine (C-X-C motif) ligand 6 (granulocyte chemotactic protein 2) | 9.29 | 9.88 | 17.31 |
| ***CFB*** | Complement factor B | 8.77 | 6.91 | 24.24 |
| ***ABCA1*** | ATP-binding cassette, sub-family A (ABC1), member 1 | 6.77 | 9.88 | 15.81 |
| ***SFRP2*** | Secreted frizzled-related protein 2 | 5.08 | 13.33 | 12.64 |
| ***CCL2*** | Chemokine (C-C motif) ligand 2 | 5.97 | 9.76 | 14.17 |
| ***SFRP4*** | Secreted frizzled-related protein 4 | 6.27 | 7.99 | 13.90 |
| ***SERPING1*** | Serpin peptidase inhibitor, clade G (C1 inhibitor), member 1 | 7.43 | 8.38 | 9.76 |
| ***PDPN*** | Podoplanin | 4.99 | 12.30 | 9.88 |
| ***CFD*** | Complement factor D (adipsin) | 8.87 | 5.84 | 10.80 |
| ***WISP2*** | WNT1 inducible signaling pathway protein 2 | 10.41 | 6.57 | 8.10 |
| ***MX1*** | myxovirus (influenza virus) resistance 1, interferon-inducible protein p78 (mouse) | 7.81 | 11.02 | 5.13 |
| ***CHI3L1*** | Chitinase 3-like 1 (cartilage glycoprotein-39) | 6.76 | 5.58 | 11.04 |
| ***COL15A1*** | Collagen, type XV, alpha 1 | 5.47 | 14.64 | 5.10 |
| ***C1R*** | Complement component 1, r subcomponent | 7.33 | 5.50 | 8.45 |
| ***ECM2*** | Extracellular matrix protein 2, female organ and adipocyte specific | 4.52 | 6.36 | 10.41 |
| ***FBLN1*** | Fibulin 1 | 5.94 | 5.37 | 9.34 |
| ***SPON1*** | Spondin 1, extracellular matrix protein | 4.72 | 7.98 | 6.98 |
| ***C1S*** | Complement component 1, s subcomponent | 5.12 | 6.59 | 6.73 |
| ***TNFAIP6*** | Tumor necrosis factor, alpha-induced protein 6 | 3.24 | 5.47 | 11.92 |
| ***EGR2*** | Early growth response 2 (Krox-20 homolog, Drosophila) | 4.78 | 10.66 | 3.15 |
| ***PTGDS*** | Prostaglandin D2 synthase 21kDa (brain) | 4.90 | 3.67 | 8.43 |
| ***CXCL2*** | Chemokine (C-X-C motif) ligand 2 | 5.53 | 6.44 | 4.21 |
| ***DHRS3*** | Dehydrogenase/reductase (SDR family) member 3 | 4.50 | 4.35 | 7.58 |
| ***FNDC1*** | Fibronectin type III domain containing 1 | 4.06 | 10.79 | 2.97 |
| ***CXCR7*** | Chemokine (C-X-C motif) receptor 7 | 4.28 | 5.41 | 5.27 |
| ***SULF2*** | Sulfatase 2 | 4.04 | 5.95 | 5.00 |
| ***IL1B*** | Interleukin 1, beta | 3.29 | 5.52 | 6.39 |
| ***IFI6*** | Interferon, alpha-inducible protein 6 | 3.51 | 6.29 | 5.04 |
| ***SOD2*** | Superoxide dismutase 2, mitochondrial, nuclear gene encoding mitochondrial protein | 3.14 | 4.43 | 7.30 |
| ***A2M*** | Alpha-2-macroglobulin | 2.79 | 6.30 | 5.70 |
| ***MME*** | Membrane metallo-endopeptidase | 4.82 | 5.62 | 3.59 |
| ***FMOD*** | Fibromodulin | 3.74 | 4.51 | 5.51 |
| ***GOLSYN*** | Syntabulin (syntaxin-interacting) (SYBU) | 3.36 | 5.68 | 4.79 |
| ***EPSTI1*** | Epithelial stromal interaction 1 (breast) | 4.48 | 4.23 | 4.80 |
| ***SVEP1*** | Sushi, von Willebrand factor type A, EGF and pentraxin domain containing 1 | 3.45 | 3.08 | 8.40 |
| ***MAN1C1*** | Mannosidase, alpha, class 1C, member 1 | 3.77 | 3.43 | 6.73 |
| ***IFITM1*** | Interferon induced transmembrane protein 1 | 3.84 | 6.77 | 3.33 |
| ***TXNIP*** | Thioredoxin interacting protein | 2.74 | 4.84 | 6.50 |
| ***PLA2G2A*** | Phospholipase A2, group IIA (platelets, synovial fluid) | 4.30 | 5.89 | 3.35 |
| ***SOX4*** | SRY (sex determining region Y)-box 4 | 2.48 | 5.70 | 5.78 |
| ***TNS3*** | Tensin 3 | 2.97 | 4.97 | 5.44 |
| ***GJA1*** | Gap junction protein, alpha 1, 43kDa | 2.80 | 3.02 | 9.46 |
| ***CXCL5*** | Chemokine (C-X-C motif) ligand 5 | 3.26 | 4.54 | 5.32 |
| ***PALM*** | Paralemmi | 3.60 | 5.49 | 3.84 |
| ***STMN2*** | Stathmin-like 2 | 3.69 | 4.77 | 3.98 |
| ***RDH10*** | Retinol dehydrogenase 10 (all-trans) | 3.81 | 3.63 | 4.85 |
| ***ABCA9*** | ATP-binding cassette, sub-family A (ABC1), member 9 | 3.49 | 2.63 | 7.30 |
| ***RARRES3*** | Retinoic acid receptor responder (tazarotene induced) 3 | 3.43 | 3.40 | 5.58 |
| ***H19*** | H19, imprinted maternally expressed transcript (non-protein coding) | 4.56 | 3.33 | 4.19 |
| ***MXRA5*** | Matrix-remodelling associated 5 | 2.88 | 2.87 | 7.59 |
| ***ABCA8*** | ATP-binding cassette, sub-family A (ABC1), member 8 | 2.65 | 2.90 | 8.10 |
| ***ALPL*** | Alkaline phosphatase, liver/bone/kidney | 3.53 | 3.71 | 4.63 |
| ***ZSWIM4*** | Zinc finger, SWIM-type containing 4 | 3.20 | 4.22 | 4.47 |
| ***OAS2*** | 2'-5'-oligoadenylate synthetase 2, 69/71kDa | 3.03 | 6.93 | 2.85 |
| ***IL6*** | Interleukin 6 (interferon, beta 2) | 3.37 | 4.75 | 3.55 |
| ***PIM1*** | Pim-1 oncogene | 2.72 | 2.73 | 6.96 |
| ***TPD52L1*** | Tumor protein D52-like 1 | 3.34 | 4.19 | 3.67 |
| ***CDKN2B*** | Cyclin-dependent kinase inhibitor 2B (p15, inhibits CDK4) | 2.35 | 4.41 | 4.84 |
| ***CTSF*** | Cathepsin F | 3.26 | 4.18 | 3.65 |
| ***SORBS2*** | Sorbin and SH3 domain containing 2 | 2.64 | 3.59 | 5.00 |
| ***NDUFA4L2*** | NADH dehydrogenase (ubiquinone) 1 alpha subcomplex, 4-like 2 | 2.22 | 4.14 | 5.03 |
| ***DDIT4*** | DNA-damage-inducible transcript 4 | 2.55 | 3.35 | 5.18 |
| ***RARRES1*** | Retinoic acid receptor responder (tazarotene induced) 1 | 2.93 | 3.08 | 4.83 |
| ***SEZ6L2*** | Seizure related 6 homolog (mouse)-like 2 | 2.38 | 4.44 | 4.04 |
| ***TRIB3*** | Tribbles homolog 3 (Drosophila) | 3.09 | 4.35 | 3.18 |
| ***SLC39A8*** | Solute carrier family 39 (zinc transporter), member 8 | 2.53 | 3.89 | 4.28 |
| ***DPYSL4*** | Dihydropyrimidinase-like 4 | 2.39 | 3.22 | 5.47 |
| ***JAM2*** | Junctional adhesion molecule 2 | 2.71 | 2.65 | 5.71 |
| ***APOD*** | Apolipoprotein D | 4.00 | 3.97 | 2.52 |
| ***SLC15A3*** | Solute carrier family 15, member 3 | 2.93 | 4.05 | 3.26 |
| ***SLC25A24*** | Solute carrier family 25 (mitochondrial carrier; phosphate carrier), member 24 | 3.01 | 3.34 | 3.78 |
| ***GBP2*** | Guanylate binding protein 2, interferon-inducible | 2.74 | 3.03 | 4.56 |
| ***C10orf10*** | Chromosome 10 open reading frame 10 | 2.75 | 2.41 | 5.67 |
| ***FGF9*** | Fibroblast growth factor 9 (glia-activating factor) | 2.47 | 2.24 | 6.75 |
| ***DRAM1*** | DNA-damage regulated autophagy modulator 1 | 2.81 | 3.80 | 3.46 |
| ***ABCA6*** | ATP-binding cassette, sub-family A (ABC1), member 6 | 2.70 | 3.11 | 4.38 |
| ***CCL8*** | Chemokine (C-C motif) ligand 8 | 2.52 | 3.75 | 3.86 |
| ***HLA-B*** | Major histocompatibility complex, class I, B | 4.03 | 2.93 | 3.08 |
| ***IFIT2*** | Interferon-induced protein with tetratricopeptide repeats 2 | 3.45 | 3.64 | 2.88 |
| ***CPE*** | Carboxypeptidase E | 2.59 | 3.93 | 3.50 |
| ***HNMT*** | Histamine N-methyltransferase | 2.46 | 2.87 | 5.00 |
| ***IFIT2*** | Interferon-induced protein with tetratricopeptide repeats 2 | 3.45 | 3.64 | 2.88 |
| ***ANGPTL2*** | Angiopoietin-like 2 | 2.74 | 3.11 | 4.12 |
| ***ACVR2A*** | Activin A receptor, type IIA | 2.33 | 3.09 | 4.66 |
| ***NCRNA00219*** | Non-protein coding RNA 219 | 2.40 | 3.90 | 3.52 |
| ***RSPO3*** | R-spondin 3 | 2.86 | 2.93 | 3.86 |
| ***SLC22A3*** | Solute carrier family 22 (extraneuronal monoamine transporter), member 3 | 2.08 | 4.55 | 3.39 |
| ***PDGFD*** | Platelet derived growth factor D | 2.39 | 3.31 | 3.99 |
| ***SLC40A1*** | Solute carrier family 40 (iron-regulated transporter), member 1 | 2.77 | 2.09 | 5.30 |
| ***FBXO32*** | F-box protein 32 | 2.21 | 3.34 | 4.15 |
| ***CXCL1*** | Chemokine (C-X-C motif) ligand 1 (melanoma growth stimulating activity, alpha) | 2.94 | 2.30 | 4.54 |
| ***CCPG1*** | Cell cycle progression 1 | 2.34 | 3.36 | 3.83 |
| ***MYH11*** | Myosin, heavy chain 11, smooth muscle | 2.03 | 3.20 | 4.53 |
| ***ADAMTS5*** | ADAM metallopeptidase with thrombospondin type 1 motif, 5 (aggrecanase-2) | 2.50 | 2.82 | 4.14 |
| ***GPNMB*** | Glycoprotein (transmembrane) nmb | 2.43 | 3.54 | 3.39 |
| ***MX2*** | Myxovirus (influenza virus) resistance 2 (mouse) | 2.43 | 4.10 | 2.89 |
| ***CRISPLD2*** | Cysteine-rich secretory protein LCCL domain containing 2 | 3.18 | 3.02 | 2.97 |
| ***LAMA4*** | Laminin, alpha 4 | 2.59 | 2.67 | 4.12 |
| ***NOX4*** | NADPH oxidase 4 | 2.12 | 3.52 | 3.82 |
| ***CYP26B1*** | Cytochrome P450, family 26, subfamily B, polypeptide 1 | 2.79 | 2.32 | 4.40 |
| ***PTPRE*** | Protein tyrosine phosphatase, receptor type, E | 2.02 | 4.20 | 3.34 |
| ***MAMDC2*** | MAM domain containing 2 | 2.32 | 2.88 | 4.24 |
| ***ECGF1*** | Endothelial cell growth factor 1 (platelet-derived) | 2.55 | 4.51 | 2.46 |
| ***VEGFA*** | Vascular endothelial growth factor A | 2.00 | 3.09 | 4.53 |
| ***GDF15*** | Growth differentiation factor 15 | 2.00 | 3.98 | 3.49 |
| ***XAF1*** | XIAP associated factor 1 | 2.17 | 4.15 | 3.06 |
| ***MSC*** | Musculin | 2.60 | 3.38 | 3.14 |
| ***MFAP4*** | Microfibrillar-associated protein 4 | 2.79 | 3.61 | 2.73 |
| ***C1QTNF1*** | C1q and tumor necrosis factor related protein 1 | 3.01 | 3.27 | 2.77 |
| ***LUM*** | Lumican | 2.51 | 3.11 | 3.45 |
| ***C3*** | Complement component 3 | 2.50 | 2.47 | 4.33 |
| ***MUC1*** | Mucin 1, cell surface associated | 2.51 | 2.54 | 4.16 |
| ***KIAA0564*** | Von Willebrand factor A domain containing 8 (VWA8) | 2.39 | 3.18 | 3.48 |
| ***PMEPA1*** | Prostate transmembrane protein, androgen induced 1 | 2.32 | 3.68 | 2.95 |
| ***TSHZ2*** | Teashirt zinc finger homeobox 2 | 2.08 | 2.51 | 4.70 |
| ***EPAS1*** | Endothelial PAS domain protein 1 | 2.71 | 3.09 | 2.88 |
| ***SERPINF1*** | Serpin peptidase inhibitor, clade F (alpha-2 antiplasmin, pigment epithelium derived factor), member 1 | 2.32 | 3.32 | 3.14 |
| ***SLIT3*** | Slit homolog 3 (Drosophila) | 2.65 | 3.38 | 2.69 |
| ***MKX*** | Mohawk homeobox | 2.93 | 2.63 | 3.12 |
| ***TUFT1*** | Tuftelin 1 | 2.09 | 2.86 | 4.02 |
| ***CH25H*** | Cholesterol 25-hydroxylase | 2.17 | 2.74 | 4.02 |
| ***DCN*** | Decorin | 2.67 | 2.19 | 4.08 |
| ***ITGA11*** | Integrin, alpha 11 | 2.20 | 3.87 | 2.77 |
| ***ANKRD37*** | Ankyrin repeat domain 37 | 3.30 | 2.37 | 2.97 |
| ***TMEM140*** | Transmembrane protein 140 | 2.78 | 2.61 | 3.08 |
| ***PLXDC2*** | Plexin domain containing 2 | 2.13 | 3.04 | 3.42 |
| ***ARHGAP28*** | Rho GTPase activating protein 28 | 2.43 | 2.06 | 4.43 |
| ***LMCD1*** | LIM and cysteine-rich domains 1 | 2.47 | 3.16 | 2.82 |
| ***MXI1*** | MAX interactor 1, dimerization protein | 2.15 | 3.21 | 3.15 |
| ***MMP11*** | Matrix metallopeptidase 11 (stromelysin 3) | 2.46 | 3.42 | 2.54 |
| ***DAAM1*** | Dishevelled associated activator of morphogenesis 1 | 2.03 | 2.60 | 3.97 |
| ***FLJ22536*** | Long intergenic non-protein coding RNA 340 | 2.05 | 3.25 | 3.12 |
| ***IFIT3*** | Interferon-induced protein with tetratricopeptide repeats 3 | 2.45 | 3.24 | 2.56 |
| ***ICAM1*** | Intercellular adhesion molecule 1 (CD54), human rhinovirus receptor | 2.10 | 2.83 | 3.41 |
| ***GAS1*** | Growth arrest-specific 1 | 2.31 | 2.69 | 3.19 |
| ***PLTP*** | Phospholipid transfer protein | 2.85 | 2.80 | 2.46 |
| ***NRCAM*** | Neuronal cell adhesion molecule | 2.41 | 2.43 | 3.34 |
| ***FCGRT*** | Fc fragment of IgG, receptor, transporter, alpha | 2.44 | 2.56 | 3.07 |
| ***POSTN*** | Periostin, osteoblast specific factor | 3.16 | 2.17 | 2.77 |
| ***BNIP3L*** | BCL2/adenovirus E1B 19kDa interacting protein 3-like | 2.22 | 2.50 | 3.39 |
| ***GAA*** | Glucosidase, alpha; acid | 2.43 | 3.08 | 2.49 |
| ***IRS2*** | Insulin receptor substrate 2 | 2.27 | 3.10 | 2.63 |
| ***HLA-F*** | Major histocompatibility complex, class I, F | 2.58 | 2.77 | 2.54 |
| ***ADARB1*** | Adenosine deaminase, RNA-specific, B1 (RED1 homolog rat) | 2.05 | 2.73 | 3.19 |
| ***MDK*** | Midkine (neurite growth-promoting factor 2) | 2.58 | 2.75 | 2.50 |
| ***CCND2*** | Cyclin D2 | 2.01 | 3.29 | 2.66 |
| ***ABCC3*** | ATP-binding cassette, sub-family C (CFTR/MRP), member 3 | 2.69 | 2.54 | 2.52 |
| ***IFIH1*** | Interferon induced with helicase C domain 1 | 2.14 | 3.15 | 2.37 |
| ***PCMTD1*** | Protein-L-isoaspartate (D-aspartate) O-methyltransferase domain containing 1 | 2.10 | 2.26 | 3.29 |
| ***THBS2*** | Thrombospondin 2 | 2.45 | 2.91 | 2.18 |
| ***DPT*** | Dermatopontin | 2.06 | 2.04 | 3.62 |
| ***THBS3*** | Thrombospondin 3 | 2.53 | 2.83 | 2.03 |
| ***NFKBIZ*** | Nuclear factor of kappa light polypeptide gene enhancer in B-cells inhibitor, zeta | 2.18 | 3.21 | 2.15 |
| ***XPNPEP2*** | X-prolyl aminopeptidase (aminopeptidase P) 2, membrane-bound | 2.03 | 2.81 | 2.53 |
| ***PTGFRN*** | Prostaglandin F2 receptor inhibitor | 2.21 | 2.35 | 2.78 |
| ***IER3*** | Immediate early response 3 | 2.16 | 2.11 | 3.16 |
| ***IL20RB*** | Interleukin 20 receptor beta | 2.18 | 2.69 | 2.41 |
| ***HSD11B1*** | Hydroxysteroid (11-beta) dehydrogenase 1 | 2.21 | 2.04 | 3.03 |
| ***LOC654096*** | PREDICTED: similar to Ovarian cancer-related protein 10-2 (OVC10-2) | 2.08 | 2.47 | 2.62 |
| ***TCTN1*** | Tectonic family member 1 | 2.01 | 2.51 | 2.62 |
| ***FAM84B*** | Family with sequence similarity 84, member B | 2.22 | 2.31 | 2.43 |
| ***FAM20A*** | Family with sequence similarity 20, member A | 2.11 | 2.19 | 2.69 |
| ***TNFAIP2*** | Tumor necrosis factor, alpha-induced protein 2 | 2.06 | 2.33 | 2.35 |
| ***PLXNB1*** | Plexin B1 | 2.29 | 2.29 | 2.30 |
| ***CREG1*** | Cellular repressor of E1A-stimulated genes 1 | 2.05 | 2.10 | 2.76 |
| ***MOCOS*** | Molybdenum cofactor sulfurase | 2.15 | 2.56 | 2.16 |
| ***ATF5*** | Activating transcription factor 5 | 2.15 | 2.29 | 2.37 |
| ***HLA-H*** | Major histocompatibility complex, class I, H (pseudogene) | 2.14 | 2.12 | 2.57 |
| ***PLSCR4*** | Phospholipid scramblase 4 | 2.00 | 2.62 | 2.03 |
| ***NNMT*** | Nicotinamide N-methyltransferase | 2.05 | 2.34 | 2.21 |
| ***KLF9*** | Kruppel-like factor 9 | 2.05 | 2.32 | 2.15 |
| ***CD302*** | CD302 molecule | 2.04 | 2.25 | 2.24 |
| ***SERPINA3*** | Serpin peptidase inhibitor, clade A (alpha-1 antiproteinase, antitrypsin), member 3 | 2.19 | 2.16 | 2.08 |
| ***CXCL16*** | Chemokine (C-X-C motif) ligand 16 | 2.04 | 2.11 | 2.26 |
| ***MIR302C*** | MicroRNA 302c | 2.03 | 2.01 | 2.23 |
| ***FTHL16*** | PREDICTED: misc_RNA | 2.08 | 2.12 | 2.06 |
| ***F2RL2*** | Coagulation factor II (thrombin) receptor-like 2 | -2.07 | -2.05 | -2.06 |
| ***ACAN*** | Aggrecan | -2.52 | -2.10 | -2.14 |
| ***ATP2B4*** | ATPase, Ca^++^ transporting, plasma membrane 4 | -2.15 | -2.45 | -2.69 |
| ***MMP1*** | Matrix metallopeptidase 1 (interstitial collagenase) | -2.13 | -2.63 | -2.59 |
| ***FLJ35409*** | PREDICTED: FLJ35409 protein | -2.14 | -2.71 | -2.65 |
| ***CDC25A*** | Cell division cycle 25A | -2.18 | -2.21 | -3.38 |
| ***ESM1*** | Endothelial cell-specific molecule 1 | -2.44 | -2.47 | -2.91 |
| ***KNTC1*** | Kinetochore associated 1 | -2.17 | -2.45 | -3.49 |
| ***HMGA1*** | High mobility group AT-hook 1 | -2.18 | -3.08 | -2.80 |
| ***GPR56*** | G protein-coupled receptor 56 | -2.31 | -3.23 | -2.57 |
| ***C11orf87*** | Chromosome 11 open reading frame 87 | -2.02 | -3.47 | -2.75 |
| ***TMEM106C*** | Transmembrane protein 106C | -2.39 | -2.85 | -2.95 |
| ***FABP5*** | Fatty acid binding protein 5 (psoriasis-associated) | -2.02 | -2.08 | -4.80 |
| ***POLQ*** | Polymerase (DNA directed), theta | -2.06 | -2.95 | -3.71 |
| ***LOC100134073*** | PREDICTED: similar to LYPDC1 protein | -2.25 | -4.42 | -2.29 |
| ***TROAP*** | Trophinin associated protein (tastin) | -2.05 | -2.79 | -4.00 |
| ***FGF5*** | Fibroblast growth factor 5 | -2.01 | -2.99 | -3.83 |
| ***SCARA3*** | Scavenger receptor class A, member 3 | -2.04 | -3.77 | -2.99 |
| ***PLEK2*** | Pleckstrin 2 | -2.09 | -3.33 | -3.33 |
| ***TMSB15A*** | Thymosin beta 15a | -2.08 | -2.64 | -4.35 |
| ***LOC646576*** | PREDICTED: hypothetical LOC646576 | -2.03 | -4.27 | -2.77 |
| ***NUF2*** | NUF2, NDC80 kinetochore complex component, homolog (S. cerevisiae) | -2.17 | -3.15 | -3.84 |
| ***CSPG4*** | Chondroitin sulfate proteoglycan 4 | -2.04 | -2.76 | -4.79 |
| ***THBD*** | Thrombomodulin | -2.02 | -2.50 | -5.53 |
| ***GPSM2*** | G-protein signaling modulator 2 (AGS3-like, C. elegans) | -2.12 | -2.94 | -4.52 |
| ***FAM180A*** | Family with sequence similarity 180, member A | -2.17 | -2.82 | -4.92 |
| ***CKS2*** | CDC28 protein kinase regulatory subunit 2 | -2.04 | -3.39 | -4.43 |
| ***CDCA2*** | Cell division cycle associated 2 | -2.30 | -3.49 | -3.95 |
| ***CCNE2*** | Cyclin E2 | -2.20 | -2.05 | -7.07 |
| ***FABP5L2*** | PREDICTED: fatty acid binding protein 5-like 2 | -2.13 | -2.63 | -5.70 |
| ***RAD51AP1*** | RAD51 associated protein 1 | -2.11 | -3.11 | -5.05 |
| ***CD24*** | CD24 molecule | -2.01 | -4.25 | -3.92 |
| ***SPC24*** | SPC24, NDC80 kinetochore complex component, homolog (S. cerevisiae) | -2.42 | -3.01 | -4.62 |
| ***STMN1*** | Stathmin 1 | -2.23 | -3.45 | -4.44 |
| ***RASD2*** | RASD family, member 2 | -2.04 | -3.21 | -5.37 |
| ***PLK4*** | Polo-like kinase 4 (Drosophila) | -2.26 | -3.25 | -4.99 |
| ***MAD2L1*** | MAD2 mitotic arrest deficient-like 1 (yeast) | -2.06 | -3.29 | -5.45 |
| ***MCM2*** | Minichromosome maintenance complex component 2 | -2.04 | -2.98 | -6.09 |
| ***UHRF1*** | Ubiquitin-like with PHD and ring finger domains 1 | -2.01 | -2.72 | -7.01 |
| ***C10orf116*** | Chromosome 10 open reading frame 116 | -2.01 | -5.83 | -3.47 |
| ***SCUBE3*** | Signal peptide, CUB domain, EGF-like 3 | -2.27 | -5.10 | -3.79 |
| ***TTK*** | TTK protein kinase | -2.09 | -4.04 | -5.31 |
| ***DEPDC1*** | DEP domain containing 1 | -2.08 | -4.13 | -5.27 |
| ***MELK*** | Maternal embryonic leucine zipper kinase | -2.13 | -3.65 | -6.06 |
| ***ASPM*** | Asp (abnormal spindle) homolog, microcephaly associated (Drosophila) | -2.32 | -4.71 | -4.58 |
| ***CENPE*** | Centromere protein E, 312kDa | -2.12 | -4.44 | -5.39 |
| ***CENPM*** | Centromere protein M | -2.04 | -3.74 | -6.78 |
| ***KIF20B*** | Kinesin family member 20B | -2.14 | -3.94 | -6.27 |
| ***FOXM1*** | Forkhead box M1 | -2.30 | -4.16 | -5.58 |
| ***HJURP*** | Holliday junction recognition protein | -2.17 | -3.97 | -6.54 |
| ***CENPA*** | Centromere protein A | -2.25 | -4.41 | -5.91 |
| ***ZWINT*** | ZW10 interactor, kinetochore protein | -2.28 | -4.48 | -5.75 |
| ***CDC2*** | Cell division cycle 2, G1 to S and G2 to M | -2.15 | -3.65 | -7.59 |
| ***KIF4A*** | Kinesin family member 4A | -2.22 | -4.35 | -6.31 |
| ***RRM2*** | Ribonucleotide reductase M2 polypeptide | -2.24 | -3.59 | -7.60 |
| ***NEFM*** | Neurofilament, medium polypeptide 150kDa | -2.22 | -3.53 | -7.92 |
| ***CRIP1*** | Cysteine-rich protein 1 (intestinal) | -2.21 | -5.90 | -5.01 |
| ***RACGAP1*** | Rac GTPase activating protein 1 | -2.02 | -4.64 | -7.01 |
| ***HHIP*** | Hedgehog interacting protein | -2.71 | -8.81 | -2.88 |
| ***CDCA8*** | Cell division cycle associated 8 | -2.24 | -4.42 | -7.17 |
| ***TACC3*** | Transforming, acidic coiled-coil containing protein 3 | -2.38 | -3.90 | -7.95 |
| ***PTTG1*** | Pituitary tumor-transforming 1 | -2.23 | -5.14 | -6.60 |
| ***CDCA3*** | Cell division cycle associated 3 | -2.01 | -5.59 | -6.73 |
| ***KIF11*** | Kinesin family member 11 | -2.20 | -4.60 | -7.52 |
| ***CENPF*** | Centromere protein F, 350/400kDa | -2.33 | -4.85 | -6.82 |
| ***GINS2*** | GINS complex subunit 2 | -2.31 | -3.43 | -9.73 |
| ***CDCA5*** | Cell division cycle associated 5 | -2.30 | -4.26 | -8.02 |
| ***CDC45L*** | Cell division cycle 45 | -2.21 | -3.34 | -11.81 |
| ***S100A4*** | S100 calcium binding protein A4 | -2.19 | -8.63 | -4.62 |
| ***BUB1*** | Budding uninhibited by benzimidazoles 1 | -2.12 | -5.62 | -7.36 |
| ***KIF23*** | Kinesin family member 23 | -2.40 | -5.18 | -7.04 |
| ***FAM64A*** | Family with sequence similarity 64, member A | -2.43 | -4.96 | -7.53 |
| ***TRIP13*** | Thyroid hormone receptor interactor 13 | -2.32 | -4.80 | -9.04 |
| ***TM4SF1*** | Transmembrane 4 L six family member 1 | -2.10 | -4.75 | -10.12 |
| ***KIAA0101*** | KIAA0101 | -2.38 | -5.34 | -8.58 |
| ***CKAP2L*** | Cytoskeleton associated protein 2-like | -2.36 | -5.09 | -10.05 |
| ***CCNB2*** | Cyclin B2 | -2.15 | -6.23 | -9.16 |
| ***PTTG3P*** | Pituitary tumor-transforming 3, pseudogene | -2.17 | -5.74 | -10.10 |
| ***BIRC5*** | Baculoviral IAP repeat containing 5 | -2.15 | -6.24 | -9.43 |
| ***AURKB*** | Aurora kinase B | -2.59 | -4.97 | -10.21 |
| ***HMMR*** | Hyaluronan-mediated motility receptor (RHAMM) | -2.31 | -5.99 | -9.58 |
| ***PBK*** | PDZ binding kinase | -2.18 | -5.78 | -10.91 |
| ***PENK*** | Proenkephalin | -2.00 | -7.32 | -9.83 |
| ***PTTG1*** | Pituitary tumor-transforming 1 | -2.10 | -6.20 | -11.31 |
| ***TPX2*** | Targeting protein for Xklp2 | -2.57 | -5.78 | -10.39 |
| ***CDKN3*** | Cyclin-dependent kinase inhibitor 3 (CDK2-associated dual specificity phosphatase) | -2.41 | -6.64 | -9.73 |
| ***NUSAP1*** | Nucleolar and spindle associated protein 1 | -2.32 | -5.42 | -12.87 |
| ***TOP2A*** | Topoisomerase (DNA) II alpha 170kDa | -2.45 | -6.52 | -10.88 |
| ***KIFC1*** | Kinesin family member C1 | -2.22 | -6.34 | -12.54 |
| ***NCAPG*** | Non-SMC condensin I complex, subunit G | -2.81 | -5.61 | -11.70 |
| ***CCNA2*** | Cyclin A2 | -2.46 | -6.66 | -11.28 |
| ***FAM83D*** | Family with sequence similarity 83, member D | -2.31 | -6.47 | -12.37 |
| ***PRC1*** | Protein regulator of cytokinesis 1 | -2.38 | -6.67 | -12.24 |
| ***AURKA*** | Aurora kinase A | -2.52 | -6.46 | -12.20 |
| ***ANLN*** | Anillin, actin binding protein | -2.33 | -6.35 | -13.93 |
| ***TK1*** | Thymidine kinase 1, soluble | -2.31 | -7.53 | -12.68 |
| ***KIF20A*** | Kinesin family member 20A | -2.29 | -8.33 | -11.94 |
| ***DLGAP5*** | Discs, large (Drosophila) homolog-associated protein 5 | -2.42 | -7.90 | -12.18 |
| ***CEP55*** | Centrosomal protein 55kDa | -2.16 | -7.40 | -19.99 |
| ***UBE2C*** | Ubiquitin-conjugating enzyme E2C | -2.43 | -7.75 | -19.66 |
| ***PODXL*** | Podocalyxin-like | -2.18 | -9.76 | -20.00 |
| ***MALL*** | Mal, T-cell differentiation protein-like | -2.38 | -17.44 | -11.76 |
| ***CDC20*** | Cell division cycle 20 | -2.67 | -9.00 | -21.48 |

Fold changes indicate gene expression differences between MSCs harvested at high cell density (CC2 MSCs, ~90% confluent) and at low cell density (CC1 MSCs, ~50% confluent). Viable second-passage AT-MSCs plated at 200 or 5,000 cells/cm^2^ were incubated for 7 days to approximately 50% confluence or 90% confluence, respectively. Positive values indicate higher expression and negative values indicate lower expression, in MSCs harvested at high cell density relative to low cell density. *p*＜0.05.
