# Supplementary material for: Gene Expression Profiles of Human Adipose Tissue-Derived Mesenchymal Stem Cells Are Modified by Cell Culture Density
Source: PLoS One. 2014 Jan 6;9(1):e83363. doi: 10.1371/journal.pone.0083363 (PMC3882209; doi:10.1371/journal.pone.0083363)
Supplement: Table S2 — Differentially expressed cytokine genes in BM-MSCs, cultured to low or high density, as determined by microarray analysis. Viable second-passage BM-MSCs plated at 200 cells/cm2 (CC1 MSCs) or 5,000 cells/cm2 (CC2 MSCs) were incubated for 7 days by which time they reached ∼50% or ∼90% confluence, respectively. After harvesting, total mRNA was isolated from pooled samples of MSCs from three donors and used in the microarray analysis. Microarray data were filtered by applying two criteria for significance, P<0.05 between culture conditions. n.d, not detected. (DOC) [file pone.0083363.s002.doc]

**Table S2. Differentially expressed cytokine genes in BM-MSCs**, cultured to low or high density, as determined by microarray analysis.

|  |  |  | **Fold Change** | |
| --- | --- | --- | --- | --- |
| **Gene symbol** | **Gene description** | **Gene classification** | **BM-MSC** | |
|  |  |  | **No.1** | **No.2** |
| ***Genes up-regulated in CC2 MSCs compared to CC1 MSCs (CC2 MSC/CC1 MSC)*** | | | | |
| ***IL1B*** | Interleukin-1 beta | Interleukin (immunoglobulin superfamily) | **1.15** | **n.d** |
| ***IL6*** | Interleukin-6 | Interleukin (Type I cytokine) | **1.69** | **2.13** |
| ***A2M*** | alpha-2-Macroglobulin | Other cytokines and related genes | **n.d** | **n.d** |
| ***MDK*** | Midkine | Heparin-binding growth factors | **n.d** | **1.16** |
| ***CXCL1*** | Chemokine (C-X-C motif) ligand 1 | Chemokine (C-X-C motif) ligands | **n.d** | **n.d** |
| ***CXCL2*** | Chemokine (C-X-C motif) ligand 2 | Chemokine (C-X-C motif) ligands | **1.61** | **1.89** |
| ***CXCL5*** | Chemokine (C-X-C motif) ligand 5 | Chemokine (C-X-C motif) ligands | **n.d** | **n.d** |
| ***CXCL6*** | Chemokine (C-X-C motif) ligand 6 | Chemokine (C-X-C motif) ligands | **n.d** | **n.d** |
| ***IL8 (CXCL8)*** | Interleukin-8 | Chemokine (C-X-C motif) ligands | **1.70** | **1.29** |
| ***CXCL16*** | Chemokine (C-X-C motif) ligand 16 | Chemokine (C-X-C motif) ligands | **2.15** | **1.60** |
| ***CCL2*** | Chemokine (C-C motif) ligand 2 | Chemokine (C-C motif) ligands | **2.54** | **2.05** |
| ***CCL8*** | Chemokine (C-C motif) ligand 8 | Chemokine (C-C motif) ligands | **n.d** | **n.d** |
| ***WISP2*** | WNT1-inducible-signaling pathway protein 2 | Connective tissue growth factors | **8.64** | **6.31** |
| ***FGF9*** | Fibroblast growth factor 9 | Fibroblast growth factors | **n.d** | **n.d** |
| ***PDGFD*** | Platelet-derived growth factor D | Platelet-derived growth factors | **1.81** | **1.57** |
| ***VEGFA*** | Vascular endothelial growth factor A | Vascular endothelial growth factors | **2.11** | **2.13** |
| ***GDF15*** | Growth differentiation factor 15 | Transforming growth factor beta | **6.69** | **5.34** |
| ***GRN*** | Granulin | Proepitheilin and PC cell-derived growth factors | **2.08** | **2.10** |

Viable second-passage BM-MSCs plated at 200 cells/cm^2^ (CC1 MSCs) or 5,000 cells/cm^2^ (CC2 MSCs) were incubated for 7 days by which time they reached ~50% or ~90% confluence, respectively. After harvesting, total mRNA was isolated from pooled samples of MSCs from three donors and used in the microarray analysis. Microarray data were filtered by applying two criteria for significance, P < 0.05 between culture conditions. *n.d*, not detected.
