# Supplementary material for: Gene Expression Profiles of Human Adipose Tissue-Derived Mesenchymal Stem Cells Are Modified by Cell Culture Density
Source: PLoS One. 2014 Jan 6;9(1):e83363. doi: 10.1371/journal.pone.0083363 (PMC3882209; doi:10.1371/journal.pone.0083363)
Supplement: Table S3 — Differentially expressed cell proliferation-associated genes in BM-MSCs, cultured to low or high density, as determined by microarray analysis. Viable second-passage BM-MSCs plated at 200 cells/cm2 (CC1 MSCs) or 5,000 cells/cm2 (CC2 MSCs) were incubated for 7 days, by which time they reached ∼50% or ∼90% confluence, respectively. After harvesting, mRNA from pooled samples of BM-MSCs was used in the microarray analysis. Microarray data were filtered by applying two criteria for significance, P<0.05 between culture conditions. n.d, not detected. (DOC) [file pone.0083363.s003.doc]

**Table S3. Differentially expressed cell proliferation-associated genes in BM-MSCs**, cultured to low or high density, as determined by microarray analysis.

|  |  |  | **Fold Change** | |
| --- | --- | --- | --- | --- |
| **Gene symbol** | **Gene description** | **Gene ontology category** | **BM-MSC** | |
|  |  |  | **No.1** | **No.2** |
| ***Genes up-regulated in CC1 MSCs compared to CC2 MSCs (CC1 MSC/CC2 MSC)*** | | | | |
| ***UBE2C*** | Ubiquitin-conjugating enzyme E2C | GO:0007067 _ mitosis | **3.61** | **4.08** |
| ***KIF20A*** | Kinesin family member 20A | GO:0000278 _ mitotic cell cycle | **4.14** | **4.49** |
| ***NCAPG*** | Non-SMC condensin I complex, subunit G | GO:0051301 _ cell division | **3.24** | **4.01** |
| ***TPX2*** | Targeting protein for Xklp2 | GO:0007067 _ mitosis | **3.48** | **3.87** |
| ***BUB1*** | Budding uninhibited by benzimidazoles 1 | GO:0051301 _ cell division GO:0000278 _ mitotic cell cycle | **3.11** | **3.52** |
| ***GINS2*** | GINS complex subunit 2 | GO:0000278 _ mitotic cell cycle | **2.71** | **2.91** |
| ***RACGAP1*** | Rac GTPase-activating protein 1 | GO:0033205 _ cell cycle cytokinesis | **2.73** | **3.06** |
| ***FOXM1*** | Forkhead box M1 | GO:0008284 _ positive regulation of cell proliferation | **3.76** | **3.50** |
| ***UHRF1*** | Ubiquitin-like with PHD and ring finger domains 1 | GO:0007049 _ cell cycle GO:0008283 _ cell proliferation | **2.29** | **1.50** |
| ***MCM2*** | Minichromosome maintenance complex component 2 | GO:0007049 _ cell cycle GO:0006260 _ DNA replication | **2.59** | **2.31** |
| ***RASD2*** | RASD family, member 2 | GO:0051897 _ positive regulation of protein kinase B signaling cascade | **1.77** | **1.56** |
| ***FGF5*** | Fibroblast growth factor 5 | GO:0051781 _ positive regulation of cell division | **2.47** | **2.62** |
| ***CDC25A*** | Cell division cycle 25A | GO:0006260 _ DNA replication GO:0000278 _ mitotic cell cycle | **2.56** | **2.31** |
| ***CCNE2*** | Cyclin E2 | GO:0000278 _ mitotic cell cycle | **2.45** | **3.00** |
| ***ESM1*** | Endothelial cell-specific molecule 1 | GO:0001558 _ regulation of cell growth | **1.81** | **1.95** |
| ***TOP2A*** | Topoisomerase (DNA) II alpha | GO:0006260 _ DNA replication GO:0000278 _ mitotic cell cycle | **3.06** | **4.09** |
| ***CDC45L*** | Cell division cycle 45 | GO:0006260 _ DNA replication GO:0000278 _ mitotic cell cycle | **2.78** | **3.46** |
| ***AURKA*** | Aurora kinase A | GO:0007049 _ cell cycle GO:0007067 _ mitosis | **4.05** | **4.07** |
| ***PRC1*** | Protein regulator of cytokinesis 1 | GO:0000910 _ cytokinesis | **3.39** | **3.67** |
| ***KIFC1*** | Kinesin family member C1 | GO:0051301 _ cell division | **4.24** | **4.32** |
| ***PTTG1*** | Pituitary tumor-transforming 1 | GO:0000278 _ mitotic cell cycle | **3.11** | **3.65** |
| ***AURKB*** | Aurora kinase B | GO:0000278 _ mitotic cell cycle | **3.81** | **3.85** |
| ***KIF23*** | Kinesin family member 23 | GO:0000278 _ mitotic cell cycle | **3.01** | **3.43** |
| ***KIF11*** | Kinesin family member 11 | GO:0007067 _ mitosis | **2.58** | **3.13** |
| ***KIF20B*** | Kinesin family member 20B | GO:0007067 _ mitosis | **2.87** | **3.28** |
| ***CENPE*** | Centromere protein E | GO:0000278 _ mitotic cell cycle | **2.59** | **2.58** |
| ***ASPM*** | Asp (abnormal spindle) homolog, microcephaly associated | GO:0007067 _ mitosis | **2.92** | **3.93** |
| ***TTK*** | TTK protein kinase | GO:0008284 _ positive regulation of cell proliferation | **3.19** | **3.91** |
| ***MAD2L1*** | mitotic arrest deficient 2-like 1 | GO:0000278 _ mitotic cell cycle | **n.d** | **n.d** |
| ***NUF2*** | NUF2, NDC80 kinetochore complex component | GO:0051301 _ cell division GO:0000278 _ mitotic cell cycle | **n.d** | **1.69** |
| ***CDC20*** | Cell division cycle 20 | GO:0000278 _ mitotic cell cycle | **4.50** | **4.68** |
| ***CCNA2*** | Cyclin A2 | GO:0000278 _ mitotic cell cycle | **4.48** | **4.74** |
| ***CCNB2*** | Cyclin B2 | GO:0000278 _ mitotic cell cycle | **3.18** | **3.60** |

Viable second-passage BM-MSCs plated at 200 cells/cm^2^ (CC1 MSCs) or 5,000 cells/cm^2^ (CC2 MSCs) were incubated for 7 days, by which time they reached ~50% or ~90% confluence, respectively. After harvesting, mRNA from pooled samples of BM-MSCs was used in the microarray analysis. Microarray data were filtered by applying two criteria for significance, P < 0.05 between culture conditions. *n.d*, not detected.
