# Supplementary material for: Gene Expression Profiles of Human Adipose Tissue-Derived Mesenchymal Stem Cells Are Modified by Cell Culture Density
Source: PLoS One. 2014 Jan 6;9(1):e83363. doi: 10.1371/journal.pone.0083363 (PMC3882209; doi:10.1371/journal.pone.0083363)
Supplement: Table S4 — Primer sequences used for RT-PCR. CCL2, Chemokine (C-C motif) ligand 2; CCL8, Chemokine (C-C motif) ligand 8; CXCL1, Chemokine (C-X-C motif) ligand 1; CXCL2, Chemokine (C-X-C motif) ligand 2; CXCL5, Chemokine (C-X-C motif) ligand 5; CXCL6, Chemokine (C-X-C motif) ligand 6; IL1B, Interleukin-1, beta; IL6, Interleukin-6; IL8, Interleukin-8; KIF20A, Kinesin family member 20A; CDC45L, Cell division cycle 45; CDC20, Cell division cycle 20; NCAPG, Non-SMC condensin I complex, subunit G; UBE2C, Ubiquitin-conjugating enzyme E2C; TOP2A, Topoisomerase II alpha; GAPDH, glyceraldehyde-3-phosphate dehydrogenase. aForward (F) and reverse (R) primers used to detect mRNA expression of the indicated targets. (DOC) [file pone.0083363.s004.doc]

**Table S4. Primer sequences used for RT-PCR.**

| **Target** | **Primer sequence ^a^** | **Product Size (bp)** |
| --- | --- | --- |
| *CCL2* | F 5’-GCAGCAAGTGTCCCAAAGAA-3’ | 202 |
|  | R 5-AACAGGGTGTCTGGGGAAAG-3’ |  |
| *CCL8* | F 5’-CTTCTGTGCCTGCTGCTCAT-3’ | 169 |
|  | R 5’-GCTTCCTTGGGACATTGGAT-3’ |  |
| *CXCL1* | F 5’-TGTGAAGGCAGGGGAATGTA-3’ | 227 |
|  | R 5’-GCCCCTTTGTTCTAAGCCAG-3’ |  |
| *CXCL2* | F 5’-CCACACTCAAGAATGGGCAG-3’ | 127 |
|  | R 5’-GCCACCAATAAGCTTCCTCC-3’ |  |
| *CXCL5* | F 5’-GTAGCCTCCCTGAAGAACGG-3’ | 132 |
|  | R 5’-CTTTTCCATGCGTGCTACTT-3’ |  |
| *CXCL6* | F 5’-GTAGCCTCCCTGAAGAACGG-3’ | 176 |
|  | R 5’-GGTCCAGGGATCTCCAGAAA-3’ |  |
| *IL1B* | F 5’-ACAGGCTGCTCTGGGATTCT-3’ | 242 |
|  | R 5’-TGAAGCCCTTGCTGTAGTGG-3’ |  |
| *IL6* | F 5’-CCAGTACCCCCAGGAGAAGA-3’ | 181 |
|  | R 5’-TTGTTTTCTGCCAGTGCCTC-3’ |  |
| *IL8* | F 5’-ACCGGAAGGAACCATCTCAC-3’ | 172 |
|  | R 5’-ATTTGGGGTGGAAAGGTTTG-3’ |  |
| *KIF20A* | F 5’-CCATCAGCAATCAGGGTCTG-3’ | 196 |
|  | R 5’-CATCAATGGTGAAGGGCTTG-3’ |  |
| *CDC45L* | F 5’-CTATACCGCAGCCAGGTTCA-3’ | 113 |
|  | R 5’-ATGGCCTGGAACTTCTGCTT-3’ |  |
| *CDC20(A)* | F 5’-CCTAGTGCTCCTGGAGAGGG-3’ | 141 |
|  | R 5’-TGCGAATGTGTCGATCACTG-3’ |  |
| *NCAPG* | F 5’-CGATGGAGGTGGAATCCTTT-3’ | 128 |
|  | R 5’-AGTAACGTGGAAGCGCACAG-3’ |  |
| *UBE2C* | F 5’-TTCCTGTCTCTCTGCCAACG-3’ | 168 |
|  | R 5’-CAATGTTGGGTTCATGAGGG-3’ |  |
| *TOP2A* | F 5’-AAGGAATCGGTACTGGGTGG-3’ | 285 |
|  | R 5’-TTCTCGGTGCCATTCAACAT-3’ |  |
| *GAPDH* | F 5’-ATCACCATCTTCCA-GGAGCG-3’ | 573 |
|  | R 5’-GTTCTTCCACCACTTCGTCC-3’ |  |

*CCL2*, *Chemokine (C-C motif) ligand 2*; *CCL8*, *Chemokine (C-C motif) ligand 8*; *CXCL1*, *Chemokine (C-X-C motif) ligand 1*; *CXCL2*, *Chemokine (C-X-C motif) ligand 2*; *CXCL5*, *Chemokine (C-X-C motif) ligand 5*; *CXCL6*, *Chemokine (C-X-C motif) ligand 6*; *IL1B*, *Interleukin-1, beta*; *IL6*, *Interleukin-6*; *IL8*, *Interleukin-8*; *KIF20A*, *Kinesin family member 20A*; *CDC45L*, *Cell division cycle 45*; *CDC20*, *Cell division cycle 20*; *NCAPG*, *Non-SMC condensin I complex, subunit G*; *UBE2C*, *Ubiquitin-conjugating enzyme E2C*; *TOP2A*, *Topoisomerase II alpha*; *GAPDH*, *glyceraldehyde-3-phosphate dehydrogenase*. ^a^Forward (F) and reverse (R) primers used to detect mRNA expression of the indicated targets.
